# P-147. Exploring the Complexity of Brucellosis: Findings from a Prospective Observational Study at AIIMS, Jodhpur

**DOI:** 10.1093/ofid/ofae631.352

**Published:** 2025-01-29

**Authors:** Santhanam Naguthevar, Akshatha Ravindra, Deepak Kumar, Naresh Kumar Midha, Durga Shankar Meena, Gopal Krishna Bohra, Vibhor Tak, Ravisekhar Gadepalli

**Affiliations:** AIIMS JODHPUR, Jodhpur, Rajasthan, India; AIIMS Jodhpur, Jodhpur, Rajasthan, India; All India Institute of Medical Sciences, Jodhpur (India), Jodhpur, Rajasthan, India; AIIMS, Jodhpur, Rajasthan, India; AIIMS, Jodhpur, Rajasthan, India; AIIMS, Jodhpur, Jodhpur, Rajasthan, India; AIIMS Jodhpur , Jodhpur, Rajasthan, India; AIIMS, Jodhpur, Jodhpur, Rajasthan, India

## Abstract

**Background:**

Brucellosis, a global health concern, is often misidentified due to diagnostic complexities. In India, its prevalence is estimated at 13.5%. This study examines its microbiological and clinical spectrum for improved management.

**Methods:**

AIIMS, Jodhpur, conducted a prospective observational study from April 2021 to March 2024, examining patient records for demographic data, clinical presentations, laboratory and microbiological findings, treatment approaches, outcomes, and excluding alternative causes of pyrexia of unknown origin (PUO)

**Results:**

Among 1142 cases analyzed, 43 were confirmed positive for brucellosis subsequent to thorough exclusion criteria. The most common age group affected by brucellosis, intriguingly, was those under 20 years, comprising 53.49% of the cases, followed by individuals aged 21 to 50 years at 30.23%, with those over 50 years representing 16.27% of the cases. The study unveiled a spectrum of clinical presentations, including 34 cases of PUO, 3 instances of spinal involvement, 5 cases with Central Nervous System (CNS) manifestations, as well as isolated occurrences of Endocarditis and Kikuchi Disease, underscoring the diverse clinical manifestations associated with brucellosis. Noteworthy clinical indicators included a high prevalence of unpasteurized milk consumption (76.74%), fever (88.37%), and joint pain (62.79%). Predominantly, B. Melitensis accounted for 30 cases, followed by B. Abortus with 11 cases and B. Suis with 2 cases.

**Conclusion:**

This study reaffirms the significant diagnostic challenge posed by brucellosis, particularly within the spectrum of PUO. Blood culture emerges as a cornerstone in the reliable diagnosis of the infection.

**Disclosures:**

**All Authors**: No reported disclosures

